# Supplementary material for: Enhancement of Cisplatin Cytotoxicity by Cu(II)–Mn(II) Schiff Base Tetradentate Complex in Human Oral Squamous Cell Carcinoma
Source: Molecules. 2020 Oct 14;25(20):4688. doi: 10.3390/molecules25204688 (PMC7587367; doi:10.3390/molecules25204688)
Supplement: Supplementary file 1 [file molecules-25-04688-s001.pdf]

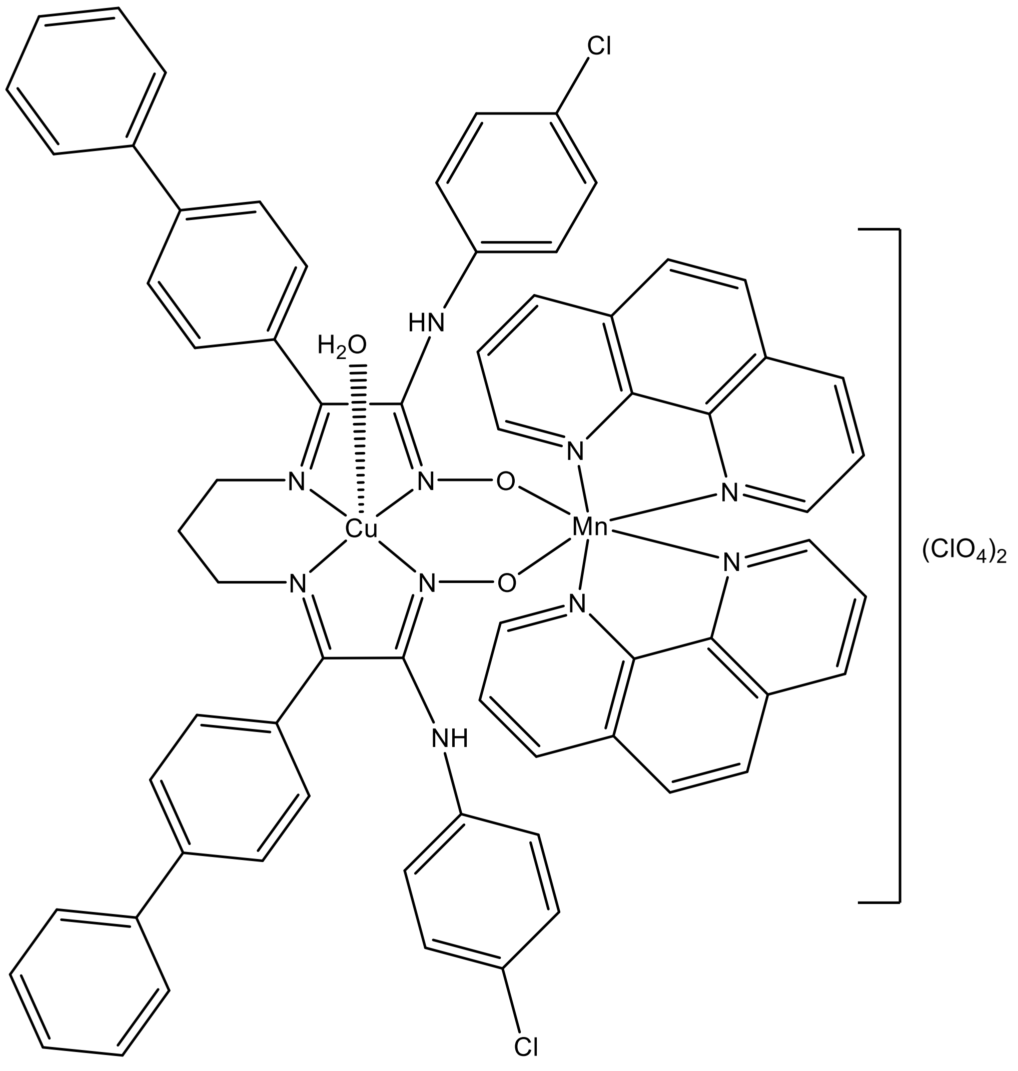

Supplementary material: [Cu(1-(Biphenyl)-2-hydroxyimino-2-(4-chloroanilino)-1-ethanone)(H_2o)Mn(phen)$_2$](Clo_4)$_2$
